# From Intervention to Innovation: Applying a Formal Implementation Strategy in Community Primary Care

**DOI:** 10.1155/2013/605757

**Published:** 2013-03-30

**Authors:** Andrea S. Wallace, Andrew L. Sussman, Mark Anthoney, Edith A. Parker

**Affiliations:** ^1^The University of Iowa College of Nursing, 330 CNB, 50 Newton Road, Iowa City, IA 52242, USA; ^2^Department of Family and Community Medicine, The University of New Mexico School of Medicine, USA; ^3^Department of Information and Technology Services, The University of Iowa, USA; ^4^Department of Community and Behavioral Health, The University of Iowa College of Public Health, USA

## Abstract

*Objective*. To describe a comprehensive strategy for implementing an effective diabetes self-management support intervention incorporating goal-setting and followup support in community health clinics (CHCs) serving vulnerable patients. *Methods*. The Replicating Effective Programs (REP) framework was applied to develop an intervention strategy. In order to create a strategy consistent with the REP framework, four CHCs engaged in an iterative process involving key-informant interviews with clinic staff, ongoing involvement of clinic staff facilitating translational efforts, feedback from national experts, and an instructional designer. *Results*. Moving through the REP process resulted in an implementation strategy that aims to facilitate commitment, communication, and change at the clinic level, as well as means of providing interactive, time-limited education about patient behavior change and support to health care providers. *Conclusion*. The REP offered a useful framework for providing guidance toward the development of a strategy to implement a diabetes self-management intervention in CHCs serving medically underserved and underrepresented patient populations.

## 1. Introduction

Effective patient self-management has been demonstrated to prevent adverse clinical outcomes from diabetes [1, 2]. While research has examined factors that influence patient receptivity and use of self-management skills, there has been less attention to the delivery of diabetes education and support in primary care settings, where most patients receive this counseling [3–5]. In fact, the quality of diabetes self-management support delivered in primary care falls short of that demonstrated to improve outcomes. Delivering even basic diabetes education is challenging to busy primary health clinics, much less providing ongoing support which addresses the many factors influencing patients' ability to make significant lifestyle changes and integrate complex tasks into their daily lives such as problem-solving, collaboration, psychosocial issues, and behavior change skills [3, 4, 6].

Collaborative goal-setting between health care providers and patients has been proposed as a strategy for providing diabetes-related self-management support in busy primary care settings [7, 8]. Because research suggests that goal-setting increases patients' self-efficacy and motivation to continue developing and maintaining self-management behaviors [9–11], goal-setting is now a common strategy in the more comprehensive Diabetes Self-Management Education curricula reimbursed by the Centers for Medicare and Medicaid Services [6, 12, 13], is an element of quality improvement efforts in primary care, and has been proposed as a measure of clinical quality [14]. However, goal-setting and followup support activities are seldom reported in primary care [8, 15], suggesting that finding cost-effective, feasible means of addressing gaps in goal-setting and followup “indicate [sic] an important area for quality improvement and diabetes self-management research” [15, page 2660].

The relative effectiveness of various delivery models for delivering diabetes self-management support (e.g., group education and individual counseling) [16–19] suggests that any number of strategies for delivering diabetes-related goal-setting and followup may be successfully tailored to the resources of individual primary care settings. However, research from implementation science suggests that numerous factors varying between individuals and organizations influence clinicians'/organizations' decisions to adopt and implement clinical interventions. These include a number of characteristics of the intervention itself such as the legitimacy of the intervention source, strength and quality of the evidence, relative advantage versus alternative solutions, adaptability, trialability, complexity, design quality and packaging, and costs [20]. Because of this, it has been argued that the gap between generation of effective interventions and widespread, sustained use in clinical practice can be addressed by strategies that consider how interventions themselves can be adapted to meet the needs of patient populations, structures, personnel, and financial incentives of individual clinic sites while also maintaining the fidelity, or consistent delivery of components necessary for an intervention to be effective [21, 22]. 

Since little is known about how best to integrate diabetes-related self-management support into the routine diabetes care provided in community (versus academic) health clinics, much less those serving vulnerable (underserved and underrepresented) populations, this study tested the usefulness of the Replicating Effective Programs (REP) framework to develop a strategy for improving diabetes self-management support, particularly goal-setting and followup support, delivered to vulnerable patient populations in community primary care settings.

## 2. Methods

This study capitalized on academic-community-based partnerships between the Iowa Center for Clinical and Translational Science, The Iowa Primary Care Association (IPCA), and four geographically diverse federally qualified community health clinics (CHCs) located across the state of Iowa. In response to preliminary survey results documenting the need for simple strategies for providing diabetes-related self-management support in the four CHCs, the development team, which consisted of a university-based research team and four clinic staff members acting as research coordinators in each of the CHCs, engaged in a participatory process in order to develop a strategy for incorporating goal-setting and followup support in community primary care settings serving vulnerable patient populations. The process involved continuous discourse within the development team, in-depth interviews with six clinic staff members working in two of the CHCs, feedback from local stakeholders and national experts, and development of materials with an instructional design professional (Table 1). The study was approved by the Community-Based Research Institutional Review Board of The University of Iowa.

### 2.1. Study Framework

The Replicating Effective Programs (REP) model, developed in 1996 by the Centers for Disease Control and Prevention (CDC) to implement HIV-AIDS behavioral and treatment programs in community-based settings, is an empirical framework combining strategies to maximize both intervention fidelity, or consistent delivery of components necessary for an intervention to be effective, and flexibility, or the ability for individual settings to adapt the intervention to their needs [21]. The REP strategy has been successfully applied to other implementation efforts including violence, substance abuse, and delinquency prevention programs as well as packaging interventions for depression care [23], suggesting its applicability beyond its initial targeted prevention efforts. Because the goal of the REP framework is to ensure successful adaptation and implementation of interventions into nonacademic, community-based settings, we believed it was particularly well-suited for efforts aiming to implement diabetes-related goal-setting and followup support in CHCs.

**Figure 1:**
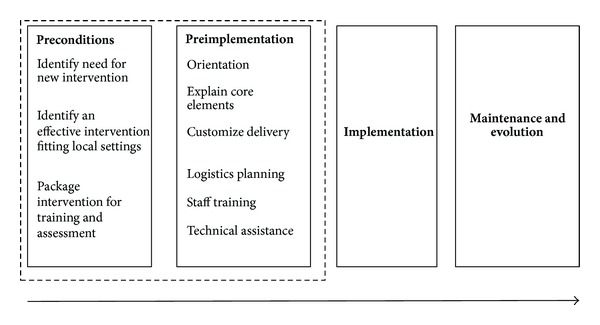
REP framework as used to plan for diabetes self-management support.

The REP model describes four key phases for researchers to consider while attempting to implement a program in nonacademic community settings: preconditions, preimplementation, implementation, and maintenance and evolution. Satisfying preconditions entails establishing the need for an intervention, identifying an intervention that addresses the needs of settings (e.g., clinics) as well as the targeted population (e.g., patients), and identifying barriers to implementation. After the needs of the setting and population have been identified, the REP model suggests implementers develop a strategy to assist sites as they attempt to use the intervention including (1) clear identification of the intervention's core elements (or factors that should not be changed in order for it to be effective) as well as elements that may help appropriately tailor the intervention to the context of individual sites (e.g., the skill sets of available staff and technological resources) and (2) an implementation package that, in everyday language, provides concrete information and resources to clinic sites about how to implement the intervention (e.g., setup procedures, underlying theory, and scripts). The next phase, preimplementation, begins with soliciting input from a community working group about the implementation strategy, which should also include training and technical assistance for clinic sites. The implementation strategy is then tested for clarity and functionality. Based on the feedback of the community working group and experiences of the preliminary test, the implementation strategy is refined and finalized for full implementation (Figure 1). This study focused on satisfying the preconditions and preimplementation phases of the REP in order to develop a strategy for implementing diabetes self-management support focused on providing goal setting and followup support, in primary care settings serving vulnerable populations. 

## 3. Results

During the fall of 2010, researchers at The University of Iowa partnered with clinicians serving as research coordinators in each of the four clinics (one physician, one nurse, and two health educators) to begin planning for an intervention to address the needs related to diabetes self-management support in the CHCs. Guided by the REP framework, this process involved weekly telephone calls and development of a research study incorporating (1) the recruitment of clinic staff to participate in key-informant interviews focusing on identifying specific needs and barriers related to diabetes-related self-management support, including goal setting and follow-up, in the clinic settings; (2) the drafting of a strategy, responsive to the needs of clinicians and staff, to overcome barriers to diabetes-related self-management support; (3) the solicitation of input from national content experts related to the implementation strategy; (4) the refinement of the strategy with the assistance of an instructional design professional.

### 3.1. Satisfying Preconditions: Data Gathering and Planning

#### 3.1.1. Identify Need for New Intervention

In 2006, The University of Iowa researchers partnered with the Iowa Primary Care Association (IPCA) and CHCs across the state to identify needs that might be addressed through academic-clinic research partnerships. Among the topics identified as problematic by primary care leadership was the quality of care being provided to patients with diabetes. To further assess this issue, quality improvement surveys were distributed to both patients and health care providers and revealed a high level of diabetes burden in these clinics, variability in the nature of diabetes-related self-management support provided by clinicians, and a lack of self-management support perceived by patients. Results of these surveys, as well as input from the IPCA, clinic leadership, and research coordinators, suggested that the four CHCs would be well-served by an intervention aiming to improve the consistency and quality of diabetes self-management support given to patients.

#### 3.1.2. Identify an Effective Intervention Fitting Local Settings

Informed with the aforementioned survey results, the university-based research team examined interventions aiming to facilitate diabetes self-management support in primary care settings. Because of its reliance on a simple strategy for establishing goal-setting and followup support, the university-based research team believed that the previously developed *Living with Diabetes* patient materials [24] and associated intervention [25, 26] would fit the needs of the four clinic settings. The Living with Diabetes (LWD) intervention consisted of low literacy patient materials coupled with a goal-setting session and two followup calls by research assistants. Both the guide and intervention were developed through a participatory process with diabetes patients and providers and were seen as responsive to the need for a simple, adaptable strategy for use in primary care. Because the materials were developed with vulnerable patient populations and focused on providing information to those with limited literacy skills in both English and Spanish, the intervention appeared well-suited for use in CHCs. An evaluation of LWD showed that the intervention resulted in successful development of goals by patients, increased self-efficacy, self-care behaviors, diabetes-related knowledge, and reductions in diabetes-related distress [25, 26]. Because the intervention involved approximately 10 minutes of patient contact per session and sessions were successfully conducted by nonclinician research associates, the research team believed it might be readily adapted to the needs of different CHC settings. The patient materials and associated information is available through the American College of Physicians Foundation (see http://www.acpfoundation.org/materials-and-guides/patient-guides/guide-products/living-with-diabetes.html).

In order to ensure that the objective of the LWD intervention met the needs of the CHCs, the university-based research team presented the intervention and associated materials to the clinic research coordinators, CHC medical directors, and IPCA medical director and staff. This process was accomplished through in-person, telephone, and email conversations as well as during in-person presentations during which the tenets of the intervention and potential logistics were discussed. The research coordinators and IPCA representatives responded enthusiastically to the potential intervention, believing it would help address the needs of their colleagues and provide a good resource for both English and Spanish speaking patients.

#### 3.1.3. Package Intervention for Training and Assessment

Between April and May 2011, six in-depth interviews were conducted with clinic staff members (2 ARNPS, 1 PA, 1 PharmD, 1 RD, and 1 MPH-Quality Director) to begin developing a strategy for implementing the LWD and in the four participating CHCs. During these hour-long interviews, participants were asked about their practice and that of their colleagues related to diabetes self-management education, including the barriers and facilitators, materials and methods they use, and their feelings about the approach of the *Living with Diabetes* guide and goal-setting strategy [24]. These interviews suggested (1) that clinicians do not use particular behavioral counseling strategies to facilitate goal-setting, (2) a belief that successfully communicating the nature of diabetes and its risks to patients is sufficient for patients to make behavior changes, and (3) that goal-setting has been a priority in the past but has not been maintained due to support staff turnover or completing duties for support staff assigned with the task. When asked about means of implementing the counseling into their practice settings, participants liked the idea of goal-setting but strongly believed that primary care providers had to play a major role in establishing and following up on patient goals rather than handing off responsibility for doing so to support staff. They all reported that the clinics were in the midst of implementing use of electronic health records (EHRs), so that incorporating goal-setting might be seen as an overwhelming task by their colleagues. However, they also reported that, unlike paper-based systems that were not sustainable, EHRs could help facilitate tracking of patient goals and follow-up. Finally, related to the training of healthcare providers in the use of the proposed LWD intervention, participants reported that the training would have to be engaging, time-limited, and presented in a way that would facilitate commitment. Participants believed that the use of goal-setting in the past was met with variable enthusiasm and foresaw that making it clear that the intervention addressed their challenges rather than adding to them would be helpful in getting providers to participate. They reported that previous interventions were influenced by those charged with implementation and believed having an esteemed colleague presenting the intervention during a group meeting of primary care providers would be most effective at winning support. 

Following the REP framework and based on the key informant interview findings, the opinions of the academic and community-based research team (i.e., the development team) and expert opinion (e.g., nationally recognized diabetes education, practice change, and implementation experts), an implementation package was created for community sites. The development of this package is described in the following section. 

### 3.2. Satisfying Preimplementation: Drafting the Implementation Package

The REP preimplementation activities include orienting settings to the intervention, explaining core elements, customizing delivery, logistics planning, staff training, and ongoing technical assistance. The core research team decided to incorporate these activities into a participatory process of developing the implementation package. The implementation package includes content to be used by a program champion—a clinician-colleague charged with the role of facilitating use of the intervention—and training materials to be used by clinicians and support staff engaged in diabetes care. The content also includes setup procedure, underlying theory and logic flow, scripts, and options for adapting the delivery of intervention core elements to local organizations in a way that does not compromise core elements, or means of ensuring intervention fidelity. Because of the desire to develop a package appropriate for larger scale dissemination efforts and because clinicians communicated the need for time limited and interactive elements, the university-based research team (after confirming the availability of the technological capacity in the CHCs) embarked on a process of organizing, tailoring, and developing content that is primarily web-based, including informative videos, checklists, interactive tutorials, and means of facilitating ongoing contact and support by the university-based researchers (Table 2). 

#### 3.2.1. Orientation

The first element to be addressed by the implementation package is to orient sites to the tenets of the intervention. A number of means of accomplishing this task were discussed. However, because clinic staff members reported a need to facilitate commitment through a group presentation, the university-based research team decided to develop a voiceover presentation which highlighted (1) that the intervention was developed by an interdisciplinary team of clinician-researchers to address the frustrations of clinicians and patients around diabetes management; (2) the basic tenets of the intervention; (3) how it might be adapted to different clinic resources. Because clinician interviews communicated the potential of clinic sites to feel overwhelmed by changes introduced by the introduction of EHRs, the development team felt it particularly important to incorporate content strongly communicating that the intent of the intervention is to *assist* clinicians in their practice, particularly with their quality improvement efforts, rather than simply providing one more thing to do. The resulting product is a hybrid video-slide show introduction that is intended to be used by program champions during a group presentation to their colleagues. In addition, content about how to resolve possible technical difficulties that could be encountered when attempting to screen the presentation is included in the program champion materials. 

#### 3.2.2. Explain Core Elements

The second element to be addressed by the implementation package is to provide an explanation of the intervention's core elements, or those key to its effectiveness. Although the LWD intervention was intended to be adapted to the needs of individual settings, the research team concluded that its core elements included (1) an initial in-person goal-setting session for which the hardcopy *Living with Diabetes* guide reinforces and provides information for patients to take home and (2) two followup support sessions. Followup support may be provided in-person or *via* telephone [25–27].

Because clinic staff members believed that supporting education and counseling by primary care providers was a key barrier to overcome but felt strongly that diabetes self-management support is most meaningful to patients if delivered by primary care providers and reinforced by other members of the health care team, the team focused on developing intervention trainings targeting primary care clinicians. However, the team also kept a broader audience in mind to facilitate use by other staff members should the clinic believe that others (e.g., nurses and health educators) should be involved with the patient goal-setting and followup process. The result is a module called Guiding Principles that is used to begin (and is used as a reference during) interactive trainings instructing those involved in diabetes care in how to engage patients in goal-setting and in the use of the hardcopy *Living with Diabetes* patient booklet. 

#### 3.2.3. Customize Delivery

According to the REP process, customizing delivery of an intervention involves tailoring it to the needs of specific patient populations and clinic settings. Because the LWD materials and goal-setting process were developed and tested in academic settings serving vulnerable patient populations, both the academic research team and clinic-based coordinators felt it was well suited for both English-and Spanish-speaking patients served by the CHCs. Further, because the goal-setting process is meant to facilitate the creation of personal goals with the help of care providers, the team felt the intervention was responsive to patients' desire for interventions customized to their personal needs as well as to the needs of primary care clinicians who have communicated a high level of frustration with behavior change counseling [24, 27] but—as reported by the clinic staff members during interviews—are without a formal strategy with which to engage and support patients. 

#### 3.2.4. Logistics Planning

A key element of the REP process is attempting to maintain an intervention's effectiveness upon dissemination to a new setting by preserving key elements of its success while allowing for adaptation to the context (e.g., staff, resources, competing priorities, and patient population) of specific clinic settings. Therefore, content in how the LWD intervention might be adapted to CHC settings in a way that maintains elements key to its effectiveness was developed for those taking on the role of program champion. The content includes (1) how an initial goal-setting session might be conducted and (2) how followup contact by primary care provider and/or clinic support staff might be accomplished during routine care for patients with diabetes. However, after feedback of national experts and stakeholders, the university-based research team recognized that the logistics of implementing the intervention also involves the fine art of facilitating organizational change. Therefore, the team sought additional resources to support program champions in the complex task of planning and implementing the intervention within their clinic settings. 

Because of its public availability and comprehensive steps for program champions to consider when implementing an intervention in a new setting, the university-based research team decided to adapt the implementation tools put forth by the TeamStepps program, which is a collective effort between the Agency of Healthcare Research and Quality and the Department of Defense to improve patient safety (see http://teamstepps.ahrq.gov/about-2cl_3.htm). The TeamStepps materials outline steps for planning and implementing a patient safety program, including sections on conducting a needs assessment, planning, training, and implementing. Details regarding planning for change, gaining leadership commitment, communicating a plan, final preparation, training, and implementation were believed most germane to this phase of the REP framework and to implementing the LWP in the CHCs. Details for each of these steps were made specific for the goal-setting intervention and patient materials and described in (1) an in-depth online presentation and (2) a checklist, both of which are intended for program champions. 

Program Champion Checklist:

(1) Plan for Change

*Completion Date *

- Map current information, processes, and available resources for diabetes self-management support in your clinic.
- Identify methods of tracking patient goals in your clinic.
  - In an EMR, other tracking systems and/or.
  - Designating one person to followup on patient goals.
- Identify teamwork deficiencies around diabetes self-management support.
  - Are there additional support needs related to functioning as a team?
  - Are there areas where *communication* between team members needs to improve before patients' behavioral goals can be tracked?
- Define the goal of your intervention as Program Champion.
  - State in one sentence what will be achieved, who will be involved (whose behavior will change), and when and where the change will occur.
  - *For example*, all primary care clinicians will begin using the Living with Diabetes counseling strategy and materials with diabetes patients, and tracking behavioral goals in a new EMR field, beginning on February 1^st^.
- Identify a team goal related to the using the Living with Diabetes Program.
  - Some examples of process goals.
    -  80% of patients with diabetes have a behavioral goal.
    - Of patients who have received the materials, 75% have received a phone call within 1 month about the goal.
    -  90% of patients with diabetes given the materials.
    - Action plans/goals set and/or progress recorded for 75% of diabetes patients seen during March.
  - Some examples of clinical outcome goals.
    - Reduction in A1Cs.
    - Improvements in patient satisfaction.
- Develop an implementation plan.
  - Identify what groups will be invited to do the online clinician training.
  - When and how will the program be introduced to the clinic (i.e., during a group meeting)?
  - Determine how long clinicians and staff will be given to complete training and, after they are introduced to the training, when the patient materials can start being used?

(2) Gain Leadership Commitment

*Completion Date*

- Inform leaders of all facets of the plan.
  - How will clinical processes be used to implement the Living with Diabetes counseling strategy?
  - How will patient's goals be tracked?
  - How the Living with Diabetes Program be introduced to the clinic?
    -  Who will be involved?
    -  How they will be trained?

(3) Communicate the Plan

*Completion Date*

- Communicate the goals of the Living with Diabetes Program during a group Meeting.
  - Make use of the online introduction provided by the research team.
- Present the detailed plan for using the Living with Diabetes Program.
  - Describe the detailed plan for using the patient materials during routine care, including concrete details of.
    - Where the materials will be located.
    - Who is involved.
    - What EMR fields (if any) are used for tracking patient's action plans.
  - Details of the online training modules outlining how to use the materials and set behavioral goals with patients.
    -  Make use of the interactive tutorial.
  - Communicate a start date.
- Supply the following.
  - Hardcopies of the patient materials.
  - Concrete examples of what needs to be done to track goals.
    -  
*For example*, if using clinical information systems, screen shots of new fields added or how to use existing fields are extremely helpful.
- Clearly identify where colleagues should go if they are having difficulty using the online training, patient materials, or tracking patient action plans.
- Elicit any final feedback.

(4) Final Preparation

*Completion Date. *Based on any additional input, refine the implementation plan regarding use of the patient materials, counseling strategy, followup of patient action plans, and training

(5) Training

*Completion Date*

- Send email invitations to targeted clinicians and staff directing them to the online training, including a reminder of.
  - Where materials are located.
  - How goals are tracked.
  - Concrete deadlines for completing the training.

(6) Implementation

*Completion Date. *Begin using the Living with Diabetes patient materials and tracking action plans!

#### 3.2.5. Staff Training

The next step in the implementation package as suggested by the REP framework is the development of the staff training or, in this case, the training of clinicians and associated staff in how to couple the *Living with Diabetes* patient guide with a goal-setting process with patients. Based on the need for short, relevant, and engaging trainings to fit into a hectic workday, the instructional designer developed an interactive, web-based tutorial based on the clinical experiences of (1) the clinician-researchers who developed the *Living with Diabetes* materials and intervention; (2) the research assistants who conducted the original intervention study using the *Living with Diabetes* patient guide [26]; (3) the research coordinators working in the CHC sites. The module, which is also informed by the intrinsic motivation model advanced by Malone and Lepper [28], interactively demonstrates how to work with low-literacy patients, common barriers to a successful goal-setting session (e.g., patients setting behavior change goals that are too large to fit into their lifestyle), and challenges clinician assumptions by using informal assessment techniques (e.g., feedback for incorrect choices). Through this simulation, clinicians practice preferred intervention strategies and, more importantly, work past common barriers to support behavior change in patients. The interactive tutorial also simulates a role-playing exercise that could be practiced during an on-site workshop. Moving through the modules takes approximately 20 minutes but, because of its interactive nature, the time required for training is highly variable and can be completed at once or incrementally. The interactivity of the tutorial also enables the development team to track clinician use of training materials and collect feedback from clinicians, with permission from the clinician, to help the team improve the web-based curriculum in future iterations. 

#### 3.2.6. Technical Assistance

According to the REP framework, the final step in the preimplementation phase is to develop means of providing ongoing assistance to clinic sites regarding the use of the intervention. This process should be proactive, in which sites are contacted routinely and prompted for questions and concerns. For next steps related to this particular study, during the implementation phase, program champions in the four clinics involved in the development process will be routinely contacted for questions, concerns, and lessons learned. In broader dissemination efforts, this process could be facilitated by having those who use the online resources disclose their name, contact information, and the dates during which they intend to begin using the intervention. In addition, while not proactive, the web-based resources display the core research team's email contact at all times. All correspondence occurring through this channel will be tracked by the research team, with the goal being a response within 48 hours.

Finally, while not a part of the definition of technical assistance as intended by the REP framework, two additional forms of needed support were identified and content was developed as a result of piloting the program champion and clinician-staff tutorials. This content relates to the possible technical problems that might be encountered while using the online resources and steps to address these should they arise and is readily accessible on the program champion and clinician-staff training websites.

## 4. Discussion

The REP offered a useful framework for providing guidance toward the development of a strategy to successfully implement a diabetes self-management support intervention incorporating goal-setting and followup support in CHCs. Using the REP to guide the development process called attention to several barriers and facilitators of implementation that may not have otherwise been explicitly addressed. That said, the process of engaging a diverse stakeholder group and soliciting in-depth opinions of clinic staff members not only added detail to the framework, but also uncovered additional areas in need of significant attention, particularly related to facilitating practice changes by program champions. 

An example of an area that might not have been emphasized without the REP framework, but one for which additional content was uncovered by the iterative development process, was the need to provide ongoing technical assistance on the web-based technology to clinic sites. According to the REP framework, technical assistance is expert support related to use of the intervention itself. To this end, a great deal of thought was put into prompting the program champions as well as tracking questions and suggestions as we move into the next REP phase of formally implementing the goal-setting and followup support intervention in clinic settings. However, an additional element of technical assistance was uncovered by the development team and stakeholders as they tested the web-based content and, at varying degrees, encountered difficulties using the technology. As a result, content providing instruction to both program champions and those using the tutorial about how to address potential technological issues was developed. 

As anticipated, interviews with clinic staff uncovered a number of areas that had to be addressed during training. First and foremost, the training needed to deliver key content in a way that was readily accessible, engaging, and time-limited. However, we were unclear whether this content should target primary care clinicians or support staff. Results of interviews with clinic staff strongly suggested the need to target primary care providers themselves, with support staff taking a secondary role in the goal-setting and followup provided to patients. However, less obvious was the amount of effort that would have to be dedicated to support those who are largely charged with the role of implementing the intervention, or the program champions. While the REP process itself is meant to facilitate commitment from a number of stakeholders, it is less explicit about means of accomplishing this at the clinic level, where the program champion is attempting to influence change. Finding methods of accomplishing the work of an intervention within busy primary care clinics is a primary challenge of any implementation effort, but interviews with clinic staff also supported a growing body of research documenting that gaining the support of those carrying out the intervention is a significant, if not the most significant, element in successfully implementing practice changes [29]. It appeared to the development team that the TeamStepps curriculum, if adapted to the particulars of the intervention, would add adequate structure to the training and support of program champions. 

Interviews with clinic staff revealed that any implementation effort in primary care is done in the extremely hectic milieu of busy practice settings. In the case of this intervention, two factors appear to work in favor of the diabetes goal-setting intervention (1) that goal-setting is seen as a measure of clinical quality for a number of payers and accrediting bodies and (2) that, while implementation of electronic medical records is currently burdensome to clinic settings, they will likely provide means of implementing and sustaining goal-setting in the CHCs. However, this speaks to the importance of steps outlined in the preconditions phase of the REP framework that help ensure that a given intervention addresses the needs of a population and that well-informed stakeholders (i.e., clinicians) believe it can be implemented.

There are numerous areas by which the process of developing the implementation strategy might be improved. The development process would have benefitted greatly from additional information regarding how to best support the role of program champion. This might have been accomplished by asking additional questions of the clinic staff members who were interviewed, or by recruiting additional participants who have been charged to making practice changes in the past. While we attempted to address this omission by incorporating and adapting content from the TeamStepps program, which is based on extensive work conducted by AHRQ and the DOD, we are yet unclear whether this element will fully address the needs of the program champions as they attempt to implement this intervention. Those examining the development process will also notice that the voice of patients is absent. While we believed that the formative work conducted during the LWD intervention itself adequately captured the opinions of patients [24], the clinic staff who were interviewed communicated that patients are likely to respond best to primary care providers emphasizing the creation of goals. Because feasibility testing of the original LWD intervention used nonclinician research assistants for the counseling process, it is unclear how patients may respond to primary care clinicians using the intervention materials and process. We will better understand whether (and how) our efforts successfully support program champions and target the appropriate clinic personnel following the testing of the implementation process. 

## 5. Conclusion

Increasing recognition of the translational gap between efficacious interventions and their widespread adaptation and use in routine clinical practice has led researchers to more systematically examine the contextual and organizational factors likely to influence implementation. In this study, we used the REP framework to guide the development of a strategy to implement a diabetes self-management intervention in community health center primary care settings. Our findings, reported here in relation to the first two REP phases of preconditions and preimplementation, demonstrate the benefit of relying on a structured approach to guide this process. Researchers considering the use of REP or other such frameworks may also consider the need to maintain flexibility as variations in contextual factors will likely influence both the approach and decisions about resource allocation. Lastly, as the evolving field of implementation science matures, it will be important for researchers to report their experiences as a way to further refine both overall protocols and specific strategies to enhance translational efforts.

## Figures and Tables

**Table 1:** Parties involved in development process.

Professional role	Study role	Those involved
University-Based Research Team	Study facilitation, data collection, and analysis	1 PhD RN 1 DNP 1 Medical Anthropologist 1 DrPH

Clinic-Based Research Coordinators	Study facilitation, ongoing input regarding implementation package	1 MD 1 RN 2 Health Educators

Clinic Staff Members	In-depth interview data regarding diabetes counseling in context of CHCs	2 ARNPS 1 PA 1 PharmD 1 RD 1 MPH-Quality Director

National Experts	Feedback on developing implementation package and revisions	2 MDs 1 ARNP 1 Health Services Researcher

Instructional Designer	Iterative development of training materials and process	1 MLS—University Technology Staff Member

**Table 2:** Elements of implementation package.

Content	Audience	Format	Purpose
Materials with clear, evidence-based steps for implementing the *Living with Diabetes* intervention	Clinical Leader, or “Program Champion”	Web-based instruction, planning checklist	Assist program champion with change process

Introduction to the *Living with Diabetes* intervention, including purpose, counseling steps, and patient materials	Clinicians and staff involved in both direct and indirect diabetes care	Web-based videos and instruction to be shared to colleagues by clinical leader during a group meeting	Facilitate buy-in

Training regarding the use of the *Living with Diabetes* intervention	Clinicians and support staff	Interactive web-based instruction	Build counseling skills in an engaging, time-limited format

Patient instruction regarding diabetes self-management	Patients with diabetes	Hardcopy of *Living with Diabetes* patient education booklet	Reinforce messages given during clinical visits in a way that is accessible to diabetes patients with limited literacy

Means of contacting the *Living with Diabetes* research team	Program Champions	Routine prompting via email, collection of lessons learned	Provide ongoing support and troubleshooting by content experts
